# Editorial: Metals in medicine

**DOI:** 10.3389/fchem.2022.979466

**Published:** 2022-07-22

**Authors:** Zhe Liu, Christian Hartinger, Nora Kulak

**Affiliations:** ^1^ Institute of Anticancer Agents Development and Theranostic Application, School of Chemistry and Chemical Engineering, Qufu Normal University, Qufu, China; ^2^ School of Chemical Sciences, The University of Auckland, Auckland, New Zealand; ^3^ Institute of Chemistry, Otto-von-Guericke-Universität Magdeburg, Magdeburg, Germany

**Keywords:** metals in medicine, anticancer agent, metallodrug, antibacterial agent, therapeutic and diagnostic agents

The field of metal-based pharmaceuticals (metallodrugs) is one of the main research areas in bioinorganic chemistry. Metallodrugs have drawn great attention for their value as therapeutic and diagnostic agents for cancer, bipolar disorder, diabetes, Alzheimer’s disease, Parkinson’s disease, and as treatments for other maladies. Platinum-based anticancer agents are some of the most widely used metallodrugs and are important components in more than 50% of cancer chemotherapies. However, limitations, such as their toxic side effects and drug resistance, initiated the development of new metallodrugs with different modes of action. Nowadays, inorganic medicinal chemists are focusing on the design of transition metal anticancer agents that might be effective against a wider range of cancers, have less side effects, have different mechanisms of action, and therefore be effective against platinum-resistant cancers and cancer types that are not responsive to current chemotherapeutics. This Research Topic aims to highlight the potential of metallodrugs and to present the reader with the latest progress of metallodrugs in the discipline of medicinal inorganic chemistry.

In this Research Topic, excellent works cover fundamental research on drug design and synthesis, structure-activity relationships, mechanistic studies, as well as important applications such as bioimaging, sensing, anticancer and antibacterial compounds.

Very recently, both half-sandwich and cyclometallated Ir^III^ complexes have drawn increasing attention as promising anticancer agents (Wang C. et al., 2017; Wang L. et al., 2021). With the help of confocal microscopy, scientists were able to observe cellular images of non-fluorescent half-sandwich Ir^III^ complexes, which significantly improved understanding towards their anticancer mechanisms (Li et al., 2018). Xu et al. report on cyclometallated Ir^III^ complexes containing natural coumarin derivatives, which showed potent anticancer activity against A549 cells with slight selectivity over normal cells. Mechanistic studies covering cell cycle arrest, intracellular ROS levels and mitochondrial membrane depolarization, etc., indicated that the iridium complex caused higher apoptosis levels in cancer cells than in normal cells. Cellular localization investigations demonstrate that the complex accumulates in lysosomes over other organelles, and then triggers lysosome-mediated apoptosis in A549 cells. The combination of strong fluorescence and anticancer activity shows that the compound has potential as a theranostic agent.

Gold complexes, similar to silver complexes, tend to have excellent antimicrobial properties. Wang et al. identified that the simple gold complex NHC^Me^-AuCl (NHC, *N*-heterocyclic carbene) has broad-spectrum antibacterial properties, and *Pseudomonas aeruginosa* (*P. aeruginosa*) had low tendency to develop drug resistance, which is one of the major issues with currently used antimicrobials. The treatment of *P. aeruginosa* with NHC^Me^-AuCl led to severe bacterial membrane crumpling and thereby disruption of the membrane integrity. Further transcriptomic analysis showed that NHC^Me^-AuCl substantially disturbed the trehalose biosynthesis and riboflavin metabolism pathways. This work demonstrates that the complex NHC^Me^-AuCl has potential as a novel antibacterial agent for future clinical application to cope with the current crisis of antimicrobial resistance.

Heterometallic complexes have been investigated to exploit the different physicochemical properties of metal centers (Hartinger et al.) and obtain compounds with uncommon modes of action. Ma et al. report on Ru(II)-Re(I) complexes as anticancer agents with the heterodimetallic compounds deriving their cytotoxicity mostly from the Re fragment. The complexes were highly stable under biologically relevant conditions and confocal microscopy studies showed that they were taken up effectively in HeLa cells. There they caused apoptosis and an arrest in the S-phase of the cell cycle. Furthermore, they induce reactive oxygen species formation while inhibiting cell migration and colony formation. This suggests that the compound type could have some potential to inhibit metastases formation.

Bleomycin acts as an antitumor agent due to its ability to produce reactive oxygen species in the presence of Fe(II) and O_2_ (Chen et al., 2008). Bai et al. report a bleomycin mimic that is an Fe(II) complex with a pentadentate ligand with DNA and albumin-binding properties. Similar to Fe(II) bleomycin, the complex can cleave DNA at µM concentrations. HSA, an important small-molecule carrier in blood, was successfully co-crystallized with the complex, and the adduct was analyzed by X-ray diffraction. High cytotoxic effects on HeLa cells were observed with the complex as well as with the HSA-Fe(II) complex adduct, whereas normal liver cells were less affected.

We hope that the reader will find in this Research Topic much new and useful information in the field of metals in medicine and that it will inspire new and unconventional approaches in this fascinating research area.
